# Sociodemographic factors affecting glycaemic control in Finnish paediatric patients with type 1 diabetes

**DOI:** 10.1002/edm2.452

**Published:** 2023-09-25

**Authors:** Riina Pironetti, Marja‐Terttu Saha, Tiina Luukkaala, Päivi Keskinen

**Affiliations:** ^1^ Department of Paediatrics Tampere University Hospital Tampere Finland; ^2^ Faculty of Medicine and Health Technology Tampere University Tampere Finland; ^3^ Research, Development and Innovation Center Tampere University Hospital Tampere Finland; ^4^ Faculty of Sciences, Health Sciences Tampere University Tampere Finland

**Keywords:** glycaemic control, paediatric, sociodemographic factors, type 1 diabetes

## Abstract

**Aims:**

Socioeconomic problems may present significant challenges when trying to reach optimal glycaemic control in paediatric patients with type 1 diabetes. We examined sociodemographic factors affecting metabolic control in patients in one of the biggest paediatric diabetes clinics in Finland.

**Methods:**

One hundred ninety‐one children (age 2–15 years; median 11 years; 47% female) with type 1 diabetes and their families were recruited during outpatient visits in the paediatric diabetes clinic of Tampere University Hospital, Finland. The participants completed a questionnaire on the family's sociodemographic background. The child's glycaemic control was assessed by both glycosylated haemoglobin (HbA1c) and time in range (TIR). Risk factors for poor (HbA1c ≥75 mmol/mol; TIR <40%) and optimal (HbA1c <53 mmol/mol; TIR ≥70%) metabolic control were searched using logistic regression analyses.

**Results:**

Living in a nuclear family, male gender, younger age and a school assistant for diabetes management were associated with the simultaneous presence of both indicators of optimal metabolic control. Poor glycaemic control, as estimated by HbA1c, was associated with lower parental education and the child's older age. Parental smoking and the child's older age were associated with poor TIR.

**Conclusion:**

This study confirms the importance of sociodemographic factors in care of Finnish paediatric patients with type 1 diabetes. Sociodemographic status markers of the family could be used as triggers to alert paediatric diabetes teams to offer more tailored care to families with new‐onset type 1 diabetes mellitus.

## Graphical abstract

Sociodemographic problems may present challenges when trying to reach optimal glycaemic control in paediatric patients with type 1 diabetes. Our study shows that in a modern Finnish healthcare system, poor glycaemic control was associated with lower parental education, parental smoking, the child's older age or having no diabetes assistant during school days according to HbA1c or TIR value. These sociodemographic status markers of poor glycaemic control of the family could be used as triggers to alert paediatric diabetes teams to offer more tailored care to families with new‐onset type 1 diabetes mellitus.

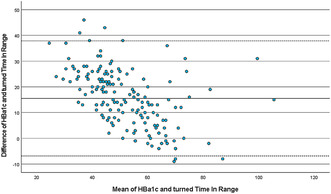

## 1 INTRODUCTION

Type 1 diabetes mellitus is one of the most common chronic diseases among paediatric population. In Finland, the incidence rate is the highest in the world: 52.2/100,000/year in population <15 years of age.^1^ The Diabetes Control and Complications Trial (DCCT) provided evidence that good metabolic control significantly decreases the risk of microvascular complications associated with type 1 diabetes.^2^ The current recommendation of The American Diabetes Association (ADA) and The International Society for Paediatric and Adolescent Diabetes (ISPAD) sets the glycaemic target for most children at HbA1c <53 mmol/mol and time in range (TIR) at >70%,^3^, ^4^ Despite recent developments in diabetes care, the majority of children with type 1 diabetes even in high‐income countries fail to achieve these glycaemic goals.^5^ In various countries, there has been increased effort to reach and maintain these metabolic goals, for example by national and international registry and intervention studies,^6^, ^7^

Sociodemographic features and socioeconomic problems may present significant challenges to reach optimal diabetes management and outcomes in children.^8^, ^9^, ^10^, ^11^, ^12^ In Finland, diabetes care is tax‐funded and organized equally for everyone. Despite a well‐organized healthcare system and a good availability of advanced diabetes technology, only one‐third of the patients reach the glycaemic targets in our clinic. Therefore, it is imperative to study the sociodemographic factors that may influence the glycaemic control in paediatric population in a modern healthcare system to be able to target the intensified healthcare support to families most in need of it, and thus help more children to achieve optimal metabolic control of their diabetes. There is paucity of data on these aspects in Finnish population.

## 2 MATERIALS AND METHODS

### 2.1 Participants and protocol

A cross‐sectional cohort study design was applied. The children with their parents were invited to participate in the study and fill out a questionnaire on a regular control visit at the paediatric outpatient diabetes clinic of Tampere University Hospital between November 2020 and October 2021. The time between visits is normally from 3 to 4 months but can for various reasons range up to 6 months. The University Hospital of Tampere is responsible of organizing the diabetes care in the whole area of Pirkanmaa District. Almost 10% of Finnish population live in Pirkanmaa District, which consists of both urban and rural areas. Participants not able to find enough time to fill the questionnaire while visiting the clinic were given a prepaid envelope to mail their answers from home.

The inclusion criteria for the study were as follows: the age of the child between 1 and 16 years, type 1 diabetes diagnosed at least 1 year before recruitment to the study, and the child being followed up at the Tampere University Hospital outpatient diabetes clinic at the moment of the recruitment. If there were several siblings with diabetes in the family, only the oldest sibling was chosen to participate. Due to the questionnaire being in Finnish, only Finnish‐speaking families were included.

An informed consent was obtained from the participants meeting the study criteria: from the caregivers and, from children 6–16 years of age, in an age‐specific manner. The study was approved by the Ethics Committee of the Pirkanmaa Hospital District.

At the time of the study, there were 429 children under the age of 16 years being followed up at the paediatric diabetes clinic of the Tampere University Hospital. Three hundred thirty‐four of them were eligible for the study. The most common reason for ineligibility was a short diabetes duration; only approximately 1% of the families are non‐Finnish speaking and excluded for this reason. 191 (57%) families agreed to participate in the study.

### 2.2 Measurements

#### 2.2.1 Data collection instrument

The questionnaire for collecting the background information of the family was developed for this study. The specific questions for the present study were as follows: the child's daycare status (home, daycare, family daycare, other), school performance (excellent, good, moderate, poor, unsatisfactory), learning difficulties (no, yes, yes ‐affirmed by a psychologist), need for extra support at school (not at all, general remedial instruction, intensified support, special support, assistant available for other reasons, assistant available due to diabetes), hobbies (specified and amount per week) and other diseases in addition to diabetes (no, yes, specified). In addition, questions on the caregivers' level of education (primary school, high school graduate, college, university of applied sciences, university, other), employment status (full‐time work, part‐time work, shift work, entrepreneur, unemployed, disability pension, irregular working hours, student) and current smoking (no, yes) were included. The family questions consisted of four items: living environment (city/concentrated area or countryside/scattered area), family structure (nuclear family, parents divorced, blended family: mother/father, joint custody, sole custody: mother/father) and number of siblings.

#### 2.2.2 Assessment of glycaemic control

The glycaemic control of the children participating in the study was assessed by both glycosylated haemoglobin (HbA1c) and TIR information, if available at the time of recruitment. HbA1c was determined using either point‐of‐care analyser (Siemens DCA Vantage) or in laboratory by a standard immunological assay at the time of recruitment. The TIR information from the previous 2 weeks was obtained from the glucose sensor data at the control visit. TIR was registered if sensor data was complete enough for this purpose as deemed by the physician. The sensors in use were FreeStyle Libre 1 or 2 (Abbott), Dexcom G6 (Dexcom Inc.) and Guardian™ 3 or 4 (Medtronic).

#### 2.2.3 Other variables

The mode of insulin treatment and diabetes duration were checked from the medical record system and the age and sex of the child participating in the study were recorded.

### 2.3 Data analysis

The characteristics of participants were described by using numbers with percentages, means with standard deviation and/or medians with interquartile range and range. The risk factors for glycated haemoglobin (HbA1c) and TIR indicating poor or optimal metabolic control were searched using logistic regression analyses. The unadjusted logistic regression models were conducted firstly for insulin delivery method (multiple‐dose injections MDI/pump), gender (girl/boy), age (continuous and categorical), school performance (excellent or good/moderate or unsatisfactory or poor/preschooler), learning difficulty (no/yes), remedial teaching at school (no/yes), type of remedial teaching at school (no/common support/intensified support/special support), assistant for other reasons than diabetes at school (no/yes), assistant for diabetes at school (no/yes). Additionally, unadjusted logistic regression models were conducted for hobbies (no/yes), number of hobbies (none/1–2/3–5), sports as hobby (no/yes), other hobbies than sports (no/yes), other disorders besides diabetes (no/yes), the highest parental educational level (vocational school/ university of applied sciences/university), parental working status (both parents working/one parent working or both parents unemployed), current parental smoking (no/at least one parent smoking), area of residence (concentrated/scattered settlement/both), number of siblings (none/1/2/3 or more siblings) and living in a nuclear family (no/yes). The risk factors with *p*‐value under .20 in unadjusted model were included in multivariable‐adjusted logistic regression models. Multivariable‐adjusted analyses were performed entering risk factors simultaneously in the adjusted models except for the combination model of good HbA1c and TIR, where univariable *p* < .20 factors were included into the model, but they were further removed if *p* ≥ .20. Results were shown using odds ratios (OR) with 95% confidence intervals (CI) and *p*‐values (*p*). We also confirmed these results using backward stepwise linear regression analysis (data not shown).

Limit values for optimal HbA1c (<53 mmol/mol) and TIR level (≥70%) were determined on the basis of the current guidelines.^3^, ^4^ The definition of poor control varies in different sources; we set this level at HbA1c 75 mmol/mol as proposed by the DCCT as the marker for poor metabolic control.^2^ The limit value for optimal TIR (≥70%) is the limit for the upper 20% of the whole TIR distribution in this study. Similarly, we chose the lowest 20% of the distribution, which is TIR 40%, as the limit indicating poor glycaemic control.

**FIGURE 1:**
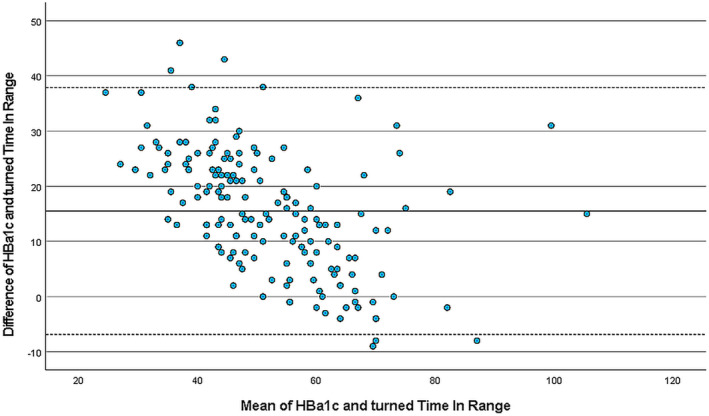
Bland–Altman plot of glycated haemoglobin (HbA1c) and Time in Range (TIR), *N* = 164. The scale of TIR was turned before plotting.

The agreement between HbA1c and TIR was shown using the Bland–Altman plot. The scale of TIR was first turned to correspond the scale of HbA1c (from optimal to poor values), that is, poor TIR value 40 corresponds after operation value 60 in Figure 1. Categorical glucose levels (for both poor and optimal HbA1c with TIR) were compared using kappa (*K*) symmetric measure of agreement.

Statistical analyses were carried out with SPSS (SPSS Inc., IBM Corp), version 26.0. The two‐sided *p* < .05 was considered statistically significant. No adjustment for multiple tests was applied and *p*‐values should be interpreted exploratorily only.

## 3 RESULTS

### 3.1 General characteristics

The characteristics of the participants included in the analysis are shown in Table 1. Altogether 191 children with the median HbA1c 59 mmol/mol were eligible for the analysis. The median percentage of TIR of the children analysed was 56%. The median duration of type 1 diabetes was 4 years.

**TABLE 1:** Characteristics of children (*N* = 191).

Gender, *n* (%)	
Girl	89 (47)
Insulin delivery method, *n* (%)	
Multiple‐dose injections	87 (46)
Insulin pump	103 (54)
Three‐times daily insulin regimen	1 (<1)
Age, median (interquartile range [IQR]; range)	11 (8–13; 2–15)
2–11 years, *n* (%)	117 (61)
12–15 years, *n* (%)	74 (39)
Time from T1DM diagnosis, median (IQR; range)	4 (2–7; 1–12)
HbA1c (mmol/mol), median (IQR; range)	59 (53–66; 39–130)
Time in range (%), median (IQR, range)	56 (44–68; 2–94)
TIR, missing values, *n* (%)	27 (14)
At least one hobby, *n* (%)	146 (76)
Other disorders besides T1DM^a^, *n* (%)	70 (37)
The highest parental educational level, *n* (%)	
University	63 (33)
University of Applied Sciences	64 (33)
Lower	64 (33)
Both parents working, *n* (%)	153 (80)
One or both parents smoking, *n* (%)	18 (9)
Number of siblings, median (range)	1 (0–8)
Nuclear family, *n* (%)	135 (71)
Concentrated settlement, *n* (%)	139 (73)

Abbreviations: HbA1c, glycosylated haemoglobin; IQR, interquartile range; T1DM, type 1 diabetes mellitus.

^a^ Other disorders: neuropsychiatric disorders, atopic dermatitis, juvenile rheumatoid arthritis, allergy, celiac disease, lactose intolerance, asthma, hearing disorder, ophthalmopathy, kidney disorder, neurological disorder, heart disorder, psychiatric disorder.

Among 191 participants, 19 children (10%, 95% confidence interval 6%–14%) had HbA1c level associated with poor metabolic control (≥75 mmol/mol) and 34 (21% of 164 children with 95% CI 15%–27%) had TIR <40%. The number of children with HbA1c indicating optimal metabolic control (<53 mmol/mol) was 47 (25%, 95% CI 18%–31%); 34 children (21% of 164 children with 95% CI 15%–27%) had TIR ≥70%. Bland–Altman plot shows the agreement between HbA1c and TIR (Figure 1). Symmetric measure Kappa‐value (*K* = 0.351) for poor values indicates a fair agreement between HbA1c and TIR measurements. Respectively, for indicators associated with optimal metabolic control the *K*‐value was 0.476, which indicates moderate agreement.^13^ TIR data were missing in 27 children (14%). Median HbA1c for those 27 children was 61 mmol/mol (IQR 55–73 mmol/mol, Range 45–130 mmol/mol). The main reasons for missing TIR data were difficulties in downloading the sensor data or the sensor not in use at the time preceding clinical visits for various reasons not investigated further.

### 3.2 Sociodemographic determinants

In unadjusted logistic regression models (Table S1), the children and adolescents in optimal glycaemic control (HbA1c <53 mmol/mol or TIR ≥70%) were found to be younger, had more often an assistant for diabetes care in classroom and needed no remedial teaching at school. They more often lived in a nuclear family, had siblings and lived in a scattered area. There was also a trend that they had multiple hobbies and, also other hobbies than sports. They tended to have both parents working and their parents were more often highly educated and non‐smoking than the parents of those in poor metabolic control. Furthermore, male gender and the use of MDI predicted better HbA1c level. In the multivariable‐adjusted analysis (Table 2), using MDI, male gender, presence of school diabetes assistant and the highest parental educational level (university level) were associated with optimal HbA1c and, respectively, no need for remedial teaching at school and presence of school diabetes assistant were associated with optimal TIR value nearly statistically significantly.

**TABLE 2:** Multivariable‐adjusted risk factors for optimal metabolic control (HbA1c <53 mmol/mol and TIR ≥70%) in children with type 1 diabetes mellitus. Risk factors *p* < .20 in the univariable‐adjusted models^a^ were included in the models. Logistic regression analyses were performed entering risk factors simultaneously in the models. Results were shown using odds ratios (OR) with 95% confidence intervals (CI) and *p*‐values (*p*).

	HbA1c <53 mmol/mol (*n* = 47, 25% of 191)			*p*	TIR ≥70% (*n* = 34, 21% of 164)			*p*
	Total	*n* (%)	OR (95% CI)		Total	*n* (%)	OR (95% CI)	
Insulin delivery method^b^								
Multiple‐dose injections	87	29 (33)	1.00		80	16 (20)	1.00	
Insulin pump	103	18 (18)	0.36 (0.16–0.79)	.011	83	18 (22)	1.50 (0.63–3.57)	.357
Gender								
Girl	89	13 (15)	1.00		78	14 (18)	1.00	
Boy	102	34 (33)	3.57 (1.56–8.19)	.003	86	20 (23)	1.57 (0.66–3.71)	.306
Age as continuous, years	191	47 (25)	1.02 (0.82–1.26)	.889	164	34 (21)	0.93 (0.72–1.19)	.555
Remedial teaching at school								.147
No	122	27 (22)	1.00		104	25 (24)	1.00	
Yes	49	11 (22)	2.08 (0.76–5.66)	.151	43	3 (7)	0.29 (0.07–1.16)	.080
Not known	20	9 (45)	8.63 (1.26–59.3)	.028	17	6 (35)	1.79 (0.23–14.0)	.578
Number of hobbies				.600				.396
None	45	7 (16)	1.00		34	3 (9)	1.00	
1–2 hobbies	123	31 (25)	1.55 (0.54–4.44)	.418	109	23 (21)	2.22 (0.56–8.85)	.259
3–5 hobbies	23	9 (39)	2.06 (0.48–8.83)	.332	21	8 (38)	3.06 (0.60–15.7)	.179
The highest parental educational level				.006				.752
Vocational school or lower	64	13 (20)	1.00		54	8 (15)	1.00	
University of Applied Sciences	64	10 (16)	0.56 (0.20–1.57)	.267	50	11 (22)	1.50 (0.49–4.57)	.475
University	63	24 (38)	2.91 (1.07–7.88)	.036	60	15 (25)	1.42 (0.47–4.28)	.533
Parental employment								
Both parents working	153	43 (28)	0.66 (0.19–2.31)	.513	134	32 (24)	2.93 (0.58–14.9)	.195
One parent working or both parents unemployed	38	4 (11)	1.00		30	2 (7)	1.00	
Child's living arrangement								
Nuclear family	135	38 (28)	1.00		119	29 (24)	1.00	
Living with one parent or with blended family	56	9 (16)	0.57 (0.22–1.48)	.247	45	5 (11)	0.57 (0.18–1.79)	.335
Assistant for T1DM at school								
No	123	25 (20)	1.00		105	15 (14)	1.00	
Yes	68	22 (32)	3.02 (1.03–8.80)	.043	59	19 (32)	2.70 (0.87–8.39)	0.086

Abbreviations: HbA1c, glycosylated haemoglobin; *n*, number of children, in good glycaemic control; T1DM, type 1 diabetes mellitus.

^a^ Risk factors tested as univariable: insulin delivery method (multiple‐dose injections/insulin pump), gender (girl/boy), age as continuous, school performance (excellent or good/moderate or poor or unsatisfactory/at daycare), learning difficulty (no/yes), remedial teaching at school (no/yes; general support/intensified support/special support), hobbies (no/yes), number of hobbies (none/1–2/3–5), sports (no/yes), other hobbies than sports (no/yes), other disorders besides T1DM (no/yes), the highest parental educational level (vocational school/university of applied sciences/university), both parents working (no/yes), at least one parent smoking (no/yes), residence (concentrated/scattered settlement), siblings (no/yes), number of siblings, living in a nuclear family (no/yes), assistant for other reasons than T1DM at school (no/yes), assistant for T1DM at school (no/yes).

^b^ One, three‐times daily insulin regimen.

In contrast, in unadjusted logistic regression models (Table S2) the study participants in poor glycaemic control (HbA1c ≥75 mmol/mol or TIR <40%) were more often teenagers, had poor school performance or learning difficulties or needed remedial teaching at school. Furthermore, they more frequently did not have an assistant for diabetes but needed an assistant for other reasons at school; their parents' highest educational level was university of applied sciences, and at least one of the parents was smoking. In the multivariable‐adjusted analysis (Table 3), older age and parental educational level university of applied sciences or lower were associated with the risk for poor HbA1c. The factors associated with poor TIR value in the multivariable‐adjusted analysis were older age and parental smoking. The presence of assistant for other reasons at school indicated poor metabolic control (HbA1c ≥75 mmol/mol) nearly statistically significantly.

**TABLE 3:** Multivariable‐adjusted risk factors for poor metabolic control (HbA1c **≥**75 mmol/mol and TIR <40%) in children with type 1 diabetes mellitus. Risk factors *p* < .20 in the univariable‐adjusted models^a^ were included in the models. Logistic regression analyses were performed entering risk factors simultaneously in the models. Results were shown using odds ratios (OR) with 95% confidence intervals (CI) and *p*‐values (*p*).

	HbA1c ≥75 mmol/mol (*n* = 19, 10% of 191)			*p*	TIR <40% (*n* = 34, 21% of 164)			*p*
	Total	*n* (%)	OR (95% CI)		Total	*n* (%)	OR (95% CI)	
Insulin delivery method^b^				.897				
Multiple‐dose injections	87	8 (9)	1.00		80	18 (23)	1.00	
Insulin pump	103	11 (11)	1.36 (0.37–4.96)	.641	83	16 (19)	0.81 (0.30–2.18)	0.674
Gender								
Girl	89	9 (10)	1.99 (0.52–7.57)	.315	78	17 (22)	1.20 (0.48–3.01)	.701
Boy	102	10 (10)	1.00		86	17 (20)	1.00	
Age as continuous (years)	191	19 (9)	2.02 (1.26–3.24)	.004	164	34 (21)	1.61 (1.18–2.20)	.002
School performance^b^				.992				
Excellent/good	146	13 (9)	1.00		125	27 (22)	1.00	
Moderate/poor/unsatisfactory	25	6 (24)	1.30 (0.24–6.95)	.756	22	6 (27)	0.39 (0.08–1.92)	.249
Preschooler	19	0 (0)	—		16	1 (6)	2.07 (0.04–110)	.721
Learning difficulties^b^								
No	162	12 (7)	1.00		140	26 (19)	1.00	
Yes	26	7 (27)	3.34 (0.44–25.3)	.244	21	7 (33)	1.93 (0.35–10.7)	.452
Remedial teaching at school^b^								
No	122	10 (8)	1.00		104	21 (20)	1.00	
Yes	49	9 (18)	0.66 (0.10–4.52)	.675	43	12 (28)	1.01 (0.24–4.23)	.985
Number of hobbies								
None	45	5 (11)	1.00		34	8 (24)	1.00	
1–2 hobbies	123	14 (11)	3.13 (0.61–16.0)	.172	109	23 (21)	1.28 (0.37–4.48)	.696
3–5 hobbies	23	0	—		21	3 (14)	0.90 (0.14–5.55)	.908
The highest parental educational level				.011				.116
Vocational school or lower	64	4 (6)	1.00		54	10 (19)	1.00	
University of applied sciences	64	12 (19)	6.76 (1.41–32.4)	.017	50	15 (30)	2.55 (0.84–7.70)	.098
University	63	3 (5)	1.08 (0.15–7.58)	.940	60	9 (15)	0.92 (0.27–3.18)	.894
Parental employment								
Both parents working	153	17 (11)	1.00	.634	134	29 (22)	1.00	.960
One parent working or both parents unemployed	38	2 (5)	0.63 (0.09–4.27)		30	5 (17)	0.97 (0.26–3.65)	
Parental smoking								
No parental smoking	173	15 (9)	1.00	.103	148	27 (18)	1.00	.039
At least one parent smoking	18	4 (22)	4.82 (0.73–32.0)		16	7 (44)	4.85 (1.09–21.6)	
Child's living arrangement								
Nuclear family	135	12 (9)	1.00	.507	119	22 (19)	1.00	.733
Living with one parent or with blended family	56	7 (13)	0.60 (0.13–2.76)		45	12 (27)	0.82 (0.27–2.52)	
Assistant for other reasons at school								
No	183	17 (9)	1.00	.058	157	31 (20)	1.00	.180
Yes	8	2 (25)	13.0 (0.92–183)		7	3 (43)	3.93 (0.53–29.1)	
Assistant for T1DM at school								
No	123	16 (13)	1.00	.608	105	26 (25)	1.00	.655
Yes	68	3 (4)	1.65 (0.24–11.3)		59	8 (14)	1.34 (0.37–4.81)	

Abbreviations: HbA1c, glycosylated haemoglobin; *n*, number of children, in poor glycaemic control; T1DM, type 1 diabetes mellitus.

^a^ Risk factors tested as univariable: insulin delivery method (multiple‐dose injections/insulin pump), gender (girl/boy), age as continuous, school performance (excellent or good/moderate or poor or unsatisfactory/at daycare), learning difficulty (no/yes), remedial teaching at school (no/yes; general support/intensified support/special support), hobbies (no/yes), number of hobbies (none/1–2/3–5), sports (no/yes), other hobbies than sports (no/yes), other disorders besides T1DM (no/yes), the highest parental educational level (vocational school/university of applied sciences/university), both parents working (no/yes), at least one parent smoking (no/yes), residence (concentrated/scattered settlement), siblings (no/yes), number of siblings, living in a nuclear family (no/yes), assistant for other reasons than T1DM at school (no/yes), assistant for T1DM at school (no/yes).

^b^ Results for missing values were not shown.

Altogether 23 (12%) of the children analysed were found to simultaneously have optimal levels of HbA1c (<53 mmol/mol) and TIR (≥70%; Table 4). In the multivariable‐adjusted analysis male gender, preschooler, living in a nuclear family and a school assistant for the diabetes associated with simultaneous presence of both indicators of good glycaemic control. The highest parental educational level (university level) was associated with the combination of both measures of good glycaemic control nearly statistically significantly.

**TABLE 4:** Univariable and multivariable‐adjusted factors associated to simultaneously optimal glycated haemoglobin (HbA1c <53 mmol/mol) and time in range (TIR ≥70%) in children with type 1 diabetes mellitus. Results for risk factors *p* < .20 in the univariable‐adjusted models^a^ were shown and they were included in the multivariable model. Multivariable‐adjusted risk factors *p* > .20 were excluded (Removed) from the final model. Logistic regression analyses were used, showing results using odds ratios (OR) with 95% confidence intervals (CI) and *p*‐values (*p*).

	HbA1c <53 mmol/mol and TIR ≥70% *n* = 23 (12%)	Everybody else *n* = 168 (88%)	HbA1c <53 mmol/mol and TIR ≥70%			
			Univariable		Multivariable	
	*n* (%)	*n* (%)	OR (95% CI)	*p*	OR (95% CI)	*p*
Gender, *n* (%)						
Girl	6 (7)	83 (93)	1.00		1.00	.009
Boy	17 (17)	85 (83)	2.77 (1.04–7.36)	.042	4.44 (1.45–13.6)	
Insulin delivery method, *n* (%)				.312	Removed	
Multiple‐dose injections	14 (16)	73 (84)	1.00			
Insulin pump	9 (9)	94 (91)	0.50 (0.20–1.22)	.127		
Three‐times daily insulin regimen	0	1 (100)	—			
Age, *n* (%)				.153	Removed	
<7 years	5 (24)	16 (76)	4.06 (0.97–17.0)	.055		
7–12 years	14 (12)	100 (88)	1.82 (0.57–5.81)	.312		
13–15 years	4 (7)	52 (93)	1.00			
School performance				.266		
Moderate/poor/unsatisfactory	2 (7)	25 (93)	1.00		1.00	
Excellent/good	16 (11)	128 (89)	1.56 (0.34–7.22)	.568	2.15 (0.37–12.4)	0.392
Preschooler	5 (26)	14 (74)	4.46 (0.76–26.1)	.097	11.0 (2.37–50.8)	0.002
Remedial teaching at school				.111	Removed	
No	15 (12)	107 (88)	1.00			
Yes	3 (6)	46 (94)	0.47 (0.13–1.69)	.244		
Not known	5 (25)	15 (75)	2.38 (0.75–7.49)	.139		
Hobbies						
No	2 (4)	43 (96)	1.00		1.00	
Yes	21 (14)	125 (86)	3.61 (0.81–16.0)	.091	3.05 (0.56–16.7)	0.199
Number of hobbies				.052	Removed	
None	2 (4)	43 (96)	1.00			
1–2 hobbies	15 (12)	108 (88)	2.99 (0.65–13.6)	.158		
3–5 hobbies	6 (26)	17 (74)	7.59 (1.39–41.4)	.019		
The highest parental educational level						
University of applied sciences or lower	12 (9)	116 (91)	1.00		1.00	
University	11 (17)	52 (83)	2.05 (0.85–4.94)	.112	2.45 (0.86–6.99)	.094
Parental employment					Not included	
Both parents working	23 (15)	130 (85)	1.00			
One parent working or both parents unemployed	0	38 (100)	—			
Parental smoking					Not included	
No parental smoking	23 (13)	150 (87)	1.00			
At least one parent smoking	0	18 (100)	—			
Child's living arrangement						
Nuclear family	21 (16)	114 (84)	4.97 (1.12–22.0)	.034	5.20 (1.03–26.3)	0.046
Living with one parent or with blended family	2 (4)	54 (96)	1.00		1.00	
Assistant for T1DM at school						
No	9 (7)	114 (93)	1.00	.009	1.00	0.001
Yes	14 (21)	54 (79)	3.28 (1.34–8.06)		7.67 (2.33–25.2)	
Other disorders besides T1DM					Removed	
No	20 (11)	161 (89)	1.00			
Yes	3 (30)	7 (70)	3.45 (0.83–14.4)	.090		

Abbreviations: HbA1c, glycosylated haemoglobin; T1DM, type 1 diabetes mellitus.

^a^ Risk factors tested as univariable: insulin delivery method (multiple‐dose injections/insulin pump/three‐times daily insulin regimen), gender (girl/boy), age as categorized, school performance (excellent or good/moderate or poor or unsatisfactory/at daycare), learning difficulty (no/yes) *p* = .547, remedial teaching at school (no/yes; general support/intensified support/special support) *p* = .244, hobbies (no/yes) *p* = .091, number of hobbies (none/1–2/3–5), sports (no/yes) *p* = .445, other hobbies than sports (no/yes) *p* = .867, other disorders besides T1DM (no/yes) *p* = .408, the highest parental educational level (university of applied sciences or lower/university), both parents working (no/yes), at least one parent smoking (no/yes), residence (concentrated/scattered settlement) *p* = .323, siblings (no/yes) *p* = .461, number of siblings, living in a nuclear family (no/yes), assistant for other reasons than T1DM at school (no/yes) *p* = .968, assistant for T1DM at school (no/yes). Risk factors, *p* < .20 as univariable, were included in the multivariable‐adjusted model, which were modelled backward stepwise. Removed = Included in multivariable model but removed due to the *p*‐value over .20. *n* = number of children, in good glycaemic control.

## 4 DISCUSSION

Our study provided evidence that, among children and adolescents with type 1 diabetes, the sociodemographic profile composed of parental education, diabetes assistant at school, parental smoking and child's living arrangement is associated with glycaemic control. To our knowledge, this is the first study including not only HbA1c but also TIR in the evaluation of sociodemographic factors affecting the glycaemic control in children and adolescents with T1DM. Even though HbA1c levels and TIR percentages measure the glycaemic control from different time periods, and TIR is related to glucose variability better than HbA1c, there was a fair agreement seen between the two measurements according to Bland–Altman plot. In addition, among the participants, there was a wide range of both HbA1c (39–130 mmol/mol) and TIR (2%–94%). Thus, the study population included representative groups of children in both optimal and poor metabolic control.

We also confirmed our results analysing the data using linear regression with backward selection for continuous HbA1c and TIR instead of grouping into poorly/well controlled. The results turned out to be quite similar (data not shown). After careful consideration and for the clarity to the clinical readers, we decided, however, to show the data by grouping the results into two extremities. As demonstrated, for example, in the DCCT/EDIC study, there was a significant difference between optimally controlled patients (HbA1c <53 mmol/mol) and poorly controlled patients (HbA1c >75 mmol/mol) in the progression of diabetes‐related complications.^14^ Similarly, defining our patients clearly in optimally controlled and poorly controlled groups highlights the factors affecting these groups more precisely. It is very important that we know which factors we focus our attention on in relation to uncontrolled patients and, on the other hand, which factors, in particular, are linked to optimal treatment balance.

In the present study, children's glycaemic control was significantly influenced by the parental educational background. The high parental education level was associated with the child's better glycaemic control. Our findings are in accordance with previous studies. Similar results have also been reported both in developed countries,^15^, ^16^ and in developing countries,^17^, ^18^, ^19^, ^20^ Highly educated parents may be keener on controlling the child's diabetes management and may be more concentrated to help their child with daily diabetes care. Another explanation could be that lower level of parental education may associate with weaker diabetes knowledge among the parents. Previous researches have demonstrated a link between low levels of diabetes knowledge of mothers and poorer glycaemic control of their children,^17^, ^20^ Accordingly, in a large French study, higher educational level of parents and diabetes knowledge were associated with better glycaemic control of their adolescents.^21^

In the multivariable‐adjusted analysis, living in a nuclear family was associated with simultaneously optimal HbA1c and TIR levels. Accordingly, Caccavale et al.^22^ studied family structure and diabetes glycaemic control status among youth. They reported that higher family density (child: parent ratio) and lower socioeconomic status (SES) were related to poorer glycaemic control via more diabetes‐related conflicts and less adherent behaviours. In addition, an association between poorer metabolic control and living in a single‐parent family has been reported,^9^, ^12^, ^23^ According to the Eurostat statistics, 8% of households with children were single‐parent families in Finland in 2020 (14% in EU).^24^ In our study cohort, 29% of the children were not living in a nuclear family, which is a remarkably higher proportion than the average. The protective mechanisms of living in a nuclear family in childhood in maintaining good metabolic control of diabetes can be diverse. For example, the economic situation might be better, enabling children to engage in hobbies more easily and improve their physical activity, the parental stress levels could be lower, diabetes management adherence perhaps better and diabetes‐related conflicts shared more equally between parents. According to a review article,^8^ living in a nuclear family improved children's HbA1c and, on the other hand, a high level of family conflicts reported by parents was associated with deteriorating of HbA1c.

In Finland, most children with T1DM who attend primary school are entitled to the help of a school diabetes assistant free of charge. The upper age limit for being eligible for such help is flexible, and its availability depends on the school and the municipality. In the present study, having a school assistant who takes care of or reminds the child of regular blood glucose monitoring, carbohydrate assessing, insulin dosing and measures to prevent hypoglycaemia was strongly associated with optimal metabolic control (both HbA1c and TIR). It is important to help small children to cope with their diabetes management during school days, as recommended, for example, by the ISPAD consensus guidelines,^4^ to be able to promote these children's academic skills. Unfortunately, not even in every primary school there is enough skilled staff to provide this important service. Being out of glycaemic target range may deteriorate the academic performance of youth with T1DM.^25^

Only 9% of the parents in our cohort were smokers. Parental smoking was associated with poor metabolic control of child's diabetes measured by TIR in our study. Previously, smoking has been associated with lower SES.^26^ Furthermore, low SES has shown to be associated with poorer metabolic control.^8^, ^9^, ^10^, ^11^

In a previous preliminary French study it was reported that the glycaemic control of children with type 1 diabetes already deteriorates during the first year following the diagnosis in families of low SES.^27^ Healthcare professionals should be able to detect familial socioeconomic and sociodemographic deprivation early so that specific educational approaches and social support could be targeted to the families most in need of them. The parental educational level, parental smoking and child's living arrangement could be considered as sociodemographic tools that may alert the healthcare professionals for the intensified, personalized interventions starting right from the diagnosis.

The multiple‐dose insulin injection therapy was associated with better glycaemic control compared to pump users. Similar trend has been observed in the benchmarking data both at our clinic and nationally before the emergence of hybrid closed‐loop pumps. In the literature, the results are often opposite when these two modes of insulin therapy are compared,^8^, ^16^ One explanation may be that our study took place before the use of hybrid closed‐loop pump systems became more common. Another reason could be that some of the children in the present study used insulin pump as ‘a rescue solution’ to improve poor metabolic control. A third reason might be suboptimal use of the insulin pumps in our study population. Whether the hybrid closed‐loop pump generation changes the course remains to be seen.

Non‐modifiable factors such as younger age and male gender were associated with better metabolic control in our study. Young age has been reported to be associated with optimal glycaemic control also previously.^16^ As diabetes management tasks among the younger children are generally managed by their parents, adherence to diabetes care is usually more intensive than among teenagers with diabetes who take care of their blood glucose control mainly by themselves. Some researchers have studied gender effect and shown that girls have higher HbA1c levels than boys,^16^, ^28^ According to a review article,^8^ the gender and age effects do not seem to be independent: no gender effect was observed before puberty or in young adults, but pubertal girls had higher HbA1c levels than boys during puberty between 13 and 21 years of age.

Some limitations should be noted in our study. Firstly, due to the nature of the study and using questionnaires with questions that may be interpreted quite intimate, some questions were left unanswered. Additionally, some questions were misinterpreted in a few cases. A suboptimal rate of participation (57%) could also be explained partly by the intimacy issue. Secondly, the small sample size might have biased the results and accordingly, the results should be interpreted with caution. Nevertheless, we consider the results to be at least indicative and valuable in the clinical practice. The reasons for not participating in the study were not inquired since it is against appropriate study ethics. Also, the glycaemic control data of those refusing to participate was not recorded. However, the cohort that participated represented well the whole patient population of the clinic in regard to glycaemic control behaviour. The median HbA1c of the study population turned out to be approximately the same as the mean HbA1c of all patients before the study period at our clinic in April 2020 (HbA1c 59.4 mmol/mol). Even though the glucose control of children in families not participating in the study did not differ from the average control in our clinic, it is possible that there may have been sociodemographic factors that could have limited the participation and thus biased the results. Also, similar unfavourable sociodemographic factors might have inhibited the proper sensor use at the time of recruitment thus leading to the missing TIR data information. Also, the study period coincided with the COVID‐19 pandemic. Our diabetes care unit could not be visited at normal intervals due to the COVID‐19 lockdown restrictions, which caused lengthening of the data collection period. The glycaemic control of some children might have been negatively influenced by the COVID‐19 lockdown restrictions by, for example decreasing participation in sports and other activities. On the contrary, in some families the presence of parents at home due to a period of remote work may have helped children to adhere better to daily tasks of their diabetes care. Due to the questionnaire being in Finnish, it is noteworthy to recognize that the study's participants exclusively consisted of Finnish‐speaking families. However, only approximately 1% of the families were non‐Finnish speaking at our clinic at the time of recruitment and were excluded for this reason. Taking into consideration the ongoing influx of immigrants to Finland and other developed European nations, it becomes imperative to examine the potential influence of this demographic factor on paediatric glycaemic control in the future.

## 5 CONCLUSIONS

In conclusion, higher sociodemographic status markers of the family, such as higher parental education level, no parental smoking and living in a nuclear family were shown to be associated with better control of type 1 diabetes in children. Whether this is due to better diabetes knowledge of the parents or better perception and adherence to diabetes control of their children remains to be studied further. In a modern Finnish healthcare system poor glycaemic control was associated with lower parental education, parental smoking, the child's older age or having no diabetes assistant during school days according to HbA1c or TIR value.

We propose that the sociodemographic factors could be used as triggers to alert paediatric diabetes teams to offer more tailored and intensified support and care to families in the need of it. As small children spend a great deal of time at school, it is imperative to have a skilled adult helping the child with diabetes management during the school day, as it appeared to be linked with better glycaemic control. This kind of help may be important especially for children with learning difficulties. These children could greatly benefit from intensified attention in diabetes management as well as from collaboration of diabetes management team and school personnel.

## AUTHOR CONTRIBUTIONS

**Riina Pironetti:** Conceptualization (equal); data curation (lead); formal analysis (equal); funding acquisition (lead); investigation (lead); methodology (equal); project administration (lead); resources (lead); software (equal); supervision (equal); validation (equal); visualization (equal); writing – original draft (lead); writing – review and editing (equal). **Marja‐Terttu Saha:** Conceptualization (equal); data curation (supporting); formal analysis (equal); funding acquisition (supporting); investigation (supporting); methodology (equal); project administration (supporting); resources (supporting); supervision (equal); validation (equal); writing – review and editing (equal). **Tiina Luukkaala:** Data curation (equal); formal analysis (equal); software (equal); visualization (supporting); writing – original draft (supporting); writing – review and editing (equal). **Päivi Keskinen:** Conceptualization (equal); data curation (supporting); formal analysis (equal); funding acquisition (supporting); investigation (supporting); methodology (equal); project administration (supporting); resources (supporting); supervision (equal); validation (equal); writing – review and editing (equal).

## FUNDING INFORMATION

Diabetes Research Foundation, Finland; Paediatric Research Foundation, Finland.

## CONFLICT OF INTEREST STATEMENT

The authors declare no potential conflict of interest.

## Supporting information


Table S1.
Table S2.

## Data Availability

The data generated and analysed during the current study are available from the corresponding author upon reasonable request.
