# Supplementary material for: Sociodemographic factors affecting glycaemic control in Finnish paediatric patients with type 1 diabetes
Source: Endocrinol Diabetes Metab. 2023 Sep 25;6(6):e452. doi: 10.1002/edm2.452 (PMC10638615; doi:10.1002/edm2.452)
Supplement: Supplementary file 1 — Table S1. Table S2. [file EDM2-6-e452-s001.docx]

## Supplementary Materials

Supplementary Table 1. Unadjusted risk factors for the optimal glycated hemoglobin (HbA1c) <53 mmol/mol and Time in Range (TIR) ≥70% in children with type 1 diabetes mellitus (T1DM). Logistic regression analysis was used, and results were shown using odds ratios (OR) with 95% confidence intervals (CI) and p-values (p).

|  | **HbA1c <53 mmol/mol** | | | | |  | **TIR ≥70 %** | | | | |  |
| --- | --- | --- | --- | --- | --- | --- | --- | --- | --- | --- | --- | --- |
|  | **(n=47, 25% of 191)** | | | | |  | **(n=34, 21% of 164)** | | | | |  |
|  | Total | n | (%) | OR | (95% CI) | p | Total | n | (%) | OR | (95% CI) | p |
| **CHILD** |  |  |  |  |  |  |  |  |  |  |  |  |
| **Insulin delivery method*** |  |  |  |  |  |  |  |  |  |  |  |  |
| Multiple-dose injections | 87 | 29 | (33) | 1.00 |  |  | 80 | 16 | (20 | 1.00 |  |  |
| Insulin pump | 103 | 18 | (18) | 0.42 | (0.21-0.83) | 0.031 | 83 | 18 | (22) | 1.11 | (0.52-2.36) | 0.791 |
| **Gender** |  |  |  |  |  |  |  |  |  |  |  |  |
| Girl | 89 | 13 | (15) | 1.00 |  |  | 78 | 14 | (18) | 1.00 |  |  |
| Boy | 102 | 34 | (33) | 2.92 | (1.42-5.99) | 0.003 | 86 | 20 | (23) | 1.39 | (0.64-2.98) | 0.404 |
| **Continuous age, years** | 191 | 47 | (25) | 0.87 | (0.78-0.98) | 0.021 | 164 | 34 | (21) | 0.84 | (0.73-0.97) | 0.014 |
| **Categorical age, years** |  |  |  |  |  | 0.114 |  |  |  |  |  | 0.250 |
| ≤6 | 21 | 9 | (43) | 1.00 |  |  | 18 | 6 | (33) | 1.00 |  |  |
| 7-12 | 114 | 27 | (24) | 0.41 | (0.15-1.09) | 0.073 | 98 | 21 | (21) | 0.55 | (0.18-1.63) | 0.277 |
| 13-15 | 56 | 11 | (20) | 0.33 | (0.11-0.97) | 0.043 | 48 | 7 | (15) | 0.34 | (0.09-1.21) | 0.096 |
| **School performance*** |  |  |  |  |  | 0.143 |  |  |  |  |  | 0.242 |
| Excellent/good | 146 | 32 | (22) | 1.00 |  |  | 125 | 26 | (21 | 1.00 |  |  |
| Moderate/poor/unsatisfactory | 25 | 6 | (24) | 1.13 | (0.41-3.05) | 0.817 | 22 | 2 | (9) | 0.38 | (0.08-1.74) | 0.212 |
| Preschooler | 19 | 9 | (47) | 3.21 | (1.20-8.56) | 0.020 | 16 | 6 | (38) | 2.29 | (0.76-6.87) | 0.141 |
| **Learning difficulty** |  |  |  |  |  |  |  |  |  |  |  |  |
| No | 162 | 38 | (24) | 1.00 |  |  | 140 | 30 | (21) | 1.00 |  |  |
| Yes | 26 | 7 | (27) | 1.20 | (0.47-3.08) | 0.701 | 21 | 2 | (10) | 0.39 | (0.08-1.75) | 0.217 |
| **Remedial teaching at school** |  |  |  |  |  | 0.096 |  |  |  |  |  | 0.033 |
| No | 122 | 27 | (22) | 1.00 |  |  | 104 | 25 | (24) | 1.00 |  |  |
| Yes | 49 | 11 | (22) | 1.02 | (0.46-2.26) | 0.964 | 43 | 3 | (7) | 0.24 | (0.06-0.83) | 0.025 |
| Not known | 20 | 9 | (45) | 2.88 | (1.08-7.66) | 0.034 | 17 | 6 | (35) | 1.72 | (0.57-5.14) | 0.328 |
| **Hobbies** |  |  |  |  |  |  |  |  |  |  |  |  |
| No | 45 | 7 | (16) | 1.00 |  |  | 34 | 3 | (9) | 1.00 |  |  |
| Yes | 146 | 40 | (27) | 2.05 | (0.84-4.96) | 0.112 | 130 | 31 | (24) | 3.24 | (0.92-11.32) | 0.066 |
| **Number of hobbies** |  |  |  |  |  | 0.109 |  |  |  |  |  | 0.045 |
| None | 45 | 7 | (16) | 1.00 |  |  | 34 | 3 | (9) | 1.00 |  |  |
| 1-2 hobbies | 123 | 31 | (25) | 1.83 | (0.74-4.51) | 0.190 | 109 | 23 | (21) | 2.76 | (0.77-9.85) | 0.117 |
| 3-5 hobbies | 23 | 9 | (39) | 3.49 | (1.09-11.2) | 0.035 | 21 | 8 | (38) | 6.36 | (1.45-27.8) | 0.014 |
| **Sports** |  |  |  |  |  |  |  |  |  |  |  |  |
| No | 72 | 19 | (27) | 1.00 |  |  | 57 | 11 | (19) | 1.00 |  |  |
| Yes | 119 | 28 | (24) | 0.86 | (0.43-1.68) | 0.657 | 107 | 23 | (22) | 1.15 | (0.51-2.26) | 0.741 |
| **Other hobby than sports** |  |  |  |  |  |  |  |  |  |  |  |  |
| No | 152 | 34 | (22) | 1.00 |  |  | 131 | 23 | (18) | 1.00 |  |  |
| Yes | 39 | 13 | (33) | 1.74 | (0.80-3.74) | 0.159 | 33 | 11 | (33) | 2.35 | (1.00-5.51) | 0.050 |
| **Other disorder besides T1DM** |  |  |  |  |  |  |  |  |  |  |  |  |
| No | 130 | 31 | (24) | 1.00 |  |  | 112 | 20 | (18) | 1.00 |  |  |
| Yes | 60 | 15 | (25) | 1.07 | (0.52-2.17) | 0.863 | 51 | 14 | (28) | 1.74 | (0.79-3.81) | 0.165 |
| **FAMILY** |  |  |  |  |  |  |  |  |  |  |  |  |
| **The highest parental educational level** |  |  |  |  |  | 0.010 |  |  |  |  |  | 0.400 |
| Vocational school | 64 | 13 | (20) | 1.00 |  |  | 54 | 8 | (15) | 1.00 |  |  |
| University of applied sciences | 64 | 10 | (16) | 0.73 | (0.29-1.80) | 0.491 | 50 | 11 | (22) | 1.62 | (0.59-4.43) | 0.346 |
| University | 63 | 24 | (38) | 2.41 | (1.09-5.33) | 0.029 | 60 | 15 | (25) | 1.92 | (0.74-4.96) | 0.180 |
| **Parental employment** |  |  |  |  |  |  |  |  |  |  |  |  |
| Both parents working | 153 | 43 | (28) | 3.32 | (1.11-9.93) | 0.022 | 134 | 32 | (24) | 4.39 | (0.99-19.46) | 0.051 |
| One parent working or both parents unemployed | 38 | 4 | (11) | 1.00 |  |  | 30 | 2 | (7) | 1.00 |  |  |
| **Parental smoking** |  |  |  |  |  |  |  |  |  |  |  |  |
| No smoking | 173 | 44 | (25) | 1.71 | (0.47-6.17) | 0.416 | 148 | 33 | (22) | 4.30 | (0.54-33.80) | 0.165 |
| At least one parent smoking | 18 | 3 | (17) | 1.00 |  |  | 16 | 1 | (6) | 1.00 |  |  |
| **Residence** |  |  |  |  |  | 0.995 |  |  |  |  |  | 0.303 |
| Concentrated settlement | 139 | 35 | (25) | 1.00 |  |  | 118 | 21 | (18) | 1.00 |  |  |
| Scattered settlement | 49 | 12 | (25) | 0.96 | (0.45-2.05) | 0.924 | 45 | 13 | (29) | 1.88 | (0.84-4.17) | 0.123 |
| Both | 3 | 0 | 0 | - |  |  | 1 | 0 | 0 | - |  |  |
| **Number of siblings** |  |  |  |  |  | 0.949 |  |  |  |  |  | 0.437 |
| None | 17 | 4 | (24) | 0.99 | (0.25-3.81) | 0.990 | 17 | 4 | (24) | 2.87 | (0.56-14.73) | 0.206 |
| 1 sibling | 86 | 23 | (27) | 1.18 | (0.48-2.86) | 0.720 | 71 | 17 | (24) | 2.94 | (0.79-10.88) | 0.107 |
| 2 siblings | 49 | 11 | (22) | 0.93 | (0.34-2.55) | 0.892 | 44 | 10 | (23) | 2.75 | (0.68-10.95) | 0.153 |
| 3 or more siblings | 38 | 9 | (24) | 1.00 |  |  | 31 | 3 | (10) | 1.00 |  |  |
| **Child’s living arrangement** |  |  |  |  |  |  |  |  |  |  |  |  |
| Nuclear family | 135 | 38 | (28) | 2.05 | (0.91-4.58) | 0.082 | 119 | 29 | (24) | 2.58 | (0.93-7.15) | 0.069 |
| Living with one parent or with blended family | 56 | 9 | (16) | 1.00 |  |  | 45 | 5 | (11) | 1.00 |  |  |
| **SUPPORT AT SCHOOL** |  |  |  |  |  |  |  |  |  |  |  |  |
| **Remedial teaching at school** |  |  |  |  |  | 0.245 |  |  |  |  |  | 0.276 |
| No | 122 | 27 | (22) | 1.00 |  |  | 104 | 25 | (24) | 1.00 |  |  |
| Common support | 23 | 4 | (17) | 0.74 | (0.23-2.37) | 0.612 | 20 | 2 | (10) | 0.35 | (0.07-1.62) | 0.180 |
| Intensified support | 17 | 5 | (29) | 1.47 | (0.47-4.53) | 0.506 | 15 | 1 | (7) | 0.23 | (0.02-1.80) | 0.160 |
| Special support | 9 | 2 | (22) | 1.01 | (0.19-5.12) | 0.995 | 8 | 0 |  | - |  |  |
| Not known | 20 | 9 | (45) | 2.88 | (1.08-7.66) | 0.034 | 17 | 6 | (35) | 1.72 | (0.57-5.14) | 0.328 |
| **Assistant for other reasons at school** |  |  |  |  |  |  |  |  |  |  |  |  |
| No | 183 | 45 | (25) | 1.00 |  |  | 157 | 33 | (21) | 1.00 |  |  |
| Yes | 8 | 2 | (25) | 1.02 | (0.20-5.25) | 0.979 | 7 | 1 | (14) | 0.63 | (0.07-5.39) | 0.670 |
| **Assistant for T1DM at school** |  |  |  |  |  |  |  |  |  |  |  |  |
| No | 123 | 25 | (20) | 1.00 |  |  | 105 | 15 | (14) | 1.00 |  |  |
| Yes | 68 | 22 | (32) | 1.88 | (0.96-3.67) | 0.067 | 59 | 19 | (32) | 2.85 | (1.32-6.17) | 0.008 |
|  |  |  |  |  |  |  |  |  |  |  |  |  |

* Missing n=1.

Supplementary Table 2. Unadjusted risk factors for the poor glycated hemoglobin (HbA1c) ≥75 mmol/mol and Time in Range (TIR) <40% in children with type 1 diabetes mellitus (T1DM). Logistic regression analysis was used, and results were shown using odds ratios (OR) with 95% confidence intervals (CI) and p-values (p).

|  | **HbA1c ≥75 mmol/mol** | | | | |  | **TIR <40 %** | | | | |  |
| --- | --- | --- | --- | --- | --- | --- | --- | --- | --- | --- | --- | --- |
|  | **(n=19, 10% of 191)** | | | | |  | **(n=34, 21% of 164)** | | | | |  |
|  | Total | n | (%) | OR | (95% CI) | p | Total | n | (%) | OR | (95% CI) | p |
| **CHILD** |  |  |  |  |  |  |  |  |  |  |  |  |
| **Insulin delivery method*** |  |  |  |  |  |  |  |  |  |  |  |  |
| Multiple-dose injections | 87 | 8 | (9) | 1.00 |  |  | 80 | 18 | (23) | 1.00 |  |  |
| Insulin pump | 103 | 11 | (11) | 1.18 | (0.45-3.08) | 0.734 | 83 | 16 | (19) | 0.82 | (0.39-1.75) | 0.613 |
| Three times daily insulin regimen | 1 | 0 |  | - |  |  | 1 | 0 |  |  |  |  |
| **Gender** |  |  |  |  |  |  |  |  |  |  |  |  |
| Girl | 89 | 9 | (10) | 1.00 |  |  | 78 | 17 | (22) | 1.00 |  |  |
| Boy | 102 | 10 | (10) | 0.97 | (0.37-2.50) | 0.943 | 86 | 17 | (20) | 0.88 | (0.41-1.88) | 0.749 |
| **Age, years** | 191 | 19 | (9) | 1.81 | (1.33-2.47) | <0.001 | 164 | 34 | (21) | 1.39 | (1.16-1.67) | <0.001 |
| **Age, years** |  |  |  |  |  | 0.002 |  |  |  |  |  | 0.011 |
| ≤6 | 21 | 0 |  | - |  |  | 18 | 1 | (6) | 0.11 | (0.01-0.88) | 0.037 |
| 7-12 | 114 | 5 | (4) | 0.14 | (0.05-0.41) | <0.001 | 98 | 16 | (16) | 0.36 | (0.16-0.79) | 0.011 |
| 13-15 | 56 | 14 | (25) | 1.00 |  |  | 48 | 17 | (35) | 1.00 |  |  |
| **School performance*** |  |  |  |  |  | 0.210 |  |  |  |  |  | 0.512 |
| Excellent/good | 146 | 13 | (9) | 1.00 |  |  | 125 | 27 | (22) | 1.00 |  |  |
| Moderate/poor/  unsatisfactory | 25 | 6 | (24) | 3.23 | (1.10-9.52) | 0.033 | 22 | 6 | (27) | 1.36 | (0.48-3.81) | 0.558 |
| Preschooler | 19 | 0 | (0) | - |  |  | 16 | 1 | (6) | 0.24 | (0.03-1.92) | 0.242 |
| Not known | 1 | 0 | (0) | - |  |  | 1 | 0 | (0) |  |  |  |
| **Learning difficulty** |  |  |  |  |  |  |  |  |  |  |  |  |
| No | 162 | 12 | (7) | 1.00 |  |  | 140 | 26 | (19) | 1.00 |  |  |
| Yes | 26 | 7 | (27) | 4.61 | (1.61-13.12) | 0.004 | 21 | 7 | (33) | 2.19 | (0.80-5.97) | 0.125 |
| **Remedial teaching at school** |  |  |  |  |  | 0.175 |  |  |  |  |  | 0.203 |
| No | 122 | 10 | (8) | 1.00 |  |  | 104 | 21 | (20) | 1.00 |  |  |
| Yes | 49 | 9 | (18) | 2.52 | (0.95-6.65) | 0.062 | 43 | 12 | (28) | 1.53 | (0.67-3.48) | 0.310 |
| Not known | 20 | 0 | (0) | - |  |  | 17 | 1 | (6) | 0.25 | (0.03-1.97) | 0.187 |
| **Hobbies** |  |  |  |  |  |  |  |  |  |  |  |  |
| No | 45 | 5 | (11) | 1.00 |  |  | 34 | 8 | (24) | 1.00 |  |  |
| Yes | 146 | 14 | (10) | 0.85 | (0.29-2.50) | 0.766 | 130 | 26 | (20) | 0.81 | (0.33-2.00) | 0.652 |
| **Number of hobbies** |  |  |  |  |  | 0.999 |  |  |  |  |  | 0.708 |
| None | 45 | 5 | (11) | 1.00 |  |  | 34 | 8 | (24) | 1.00 |  |  |
| 1-2 hobbies | 123 | 14 | (11) | 1.03 | (0.35-3.04) | 0.961 | 109 | 23 | (21) | 0.87 | (0.35-2.17) | 0.764 |
| 3-5 hobbies | 23 | 0 |  | - |  |  | 21 | 3 | (14) | 0.54 | (0.13-2.33) | 0.409 |
| **Sports** |  |  |  |  |  |  |  |  |  |  |  |  |
| No | 72 | 6 | (8) | 1.00 |  |  | 57 | 12 | (21) | 1.00 |  |  |
| Yes | 119 | 13 | (11) | 1.35 | (0.48-3.72) | 0.563 | 107 | 22 | (21) | 0.97 | (0.44-2.14) | 0.941 |
| **Other hobby than sports** |  |  |  |  |  |  |  |  |  |  |  |  |
| No | 152 | 18 | (12) | 1.00 |  |  | 131 | 29 | (22) | 1.00 |  |  |
| Yes | 39 | 1 | (3) | 0.20 | (0.02-1.52) | 0.118 | 33 | 5 | (15) | 0.63 | (0.22-1.77) | 0.379 |
| **Other disorder besides T1DM** |  |  |  |  |  |  |  |  |  |  |  |  |
| No | 130 | 14 | (11) | 1.00 |  |  | 112 | 24 | (21) | 1.00 |  |  |
| Yes | 60 | 5 | (8) | 0.75 | (0.25-2.20) | 0.604 | 51 | 10 | (20) | 0.89 | (0.39-2.04) | 0.791 |
| **FAMILY** |  |  |  |  |  |  |  |  |  |  |  |  |
| **The highest parental educational level** |  |  |  |  |  | 0.024 |  |  |  |  |  | 0.145 |
| Vocational school | 64 | 4 | (6) | 1.00 |  |  | 54 | 10 | (19) | 1.00 |  |  |
| University of applied sciences | 64 | 12 | (19) | 3.46 | (1.05-11.39) | 0.041 | 50 | 15 | (30) | 1.89 | (0.75-4.71) | 0.174 |
| University | 63 | 3 | (5) | 0.75 | (0.16-3.50) | 0.714 | 60 | 9 | (15) | 0.78 | (0.28-2.08) | 0.615 |
| **Parental employment** |  |  |  |  |  |  |  |  |  |  |  |  |
| Both parents working | 153 | 17 | (11) | 2.25 | (0.49-10.19) | 0.293 | 134 | 29 | (22) | 1.38 | (0.48-3.93) | 0.545 |
| One parent working or both parents unemployed | 38 | 2 | (5) | 1.00 |  |  | 30 | 5 | (17) | 1.00 |  |  |
| **Parental smoking** |  |  |  |  |  |  |  |  |  |  |  |  |
| No smoking | 173 | 15 | (9) | 1.00 |  |  | 148 | 27 | (18) | 1.00 |  |  |
| At least one parent smoking | 18 | 4 | (22) | 3.01 | (0.88-10.3) | 0.079 | 16 | 7 | (44) | 3.49 | (1.19-10.2) | 0.022 |
| **Residence** |  |  |  |  |  |  |  |  |  |  |  |  |
| Concentrated settlement | 139 | 12 | (9) | 1.00 |  |  | 118 | 24 | (20) | 1.00 |  |  |
| Scattered settlement | 49 | 7 | (14) | 1.76 | (0.65-4.77) | 0.264 | 45 | 10 | (22) | 1.12 | (0.48-2.58) | 0.791 |
| Both | 3 | 0 | (0) |  |  |  | 1 | 0 | (0) |  |  |  |
| **Number of siblings** |  |  |  |  |  | 0.597 |  |  |  |  |  | 0.220 |
| None | 17 | 2 | (12) | 1.13 | (0.18-6.88) | 0.892 | 17 | 4 | (24) | 0.65 | (0.16-2.49) | 0.526 |
| 1 sibling | 86 | 6 | (7) | 0.64 | (0.16-2.40) | 0.506 | 71 | 10 | (14) | 0.34 | (0.12-0.94) | 0.038 |
| 2 siblings | 49 | 7 | (14) | 1.42 | (0.38-5.25) | 0.602 | 44 | 10 | (23) | 0.62 | (0.22-1.73) | 0.360 |
| 3 or more siblings | 38 | 4 | (11) | 1.00 |  |  | 31 | 10 | (32) | 1.00 |  |  |
| **Child’s living arrangement** |  |  |  |  |  |  |  |  |  |  |  |  |
| Nuclear family | 135 | 12 | (9) | 0.68 | (0.25-1.84) | 0.450 | 119 | 22 | (19) | 0.62 | (0.28-1.40) | 0.251 |
| Living with one parent or with blended family | 56 | 7 | (13) | 1.00 |  |  | 45 | 12 | (27) | 1.00 |  |  |
| **SUPPORT AT SCHOOL** |  |  |  |  |  |  |  |  |  |  |  |  |
| **Remedial teaching at school** |  |  |  |  |  | 0.071 |  |  |  |  |  | 0.450 |
| No | 122 | 10 | (8) | 1.00 |  |  | 104 | 21 | (20) | 1.00 |  |  |
| Common support | 23 | 3 | (13) | 1.68 | (0.42-6.65) | 0.460 | 20 | 5 | (25) | 1.32 | (0.43-4.04) | 0.629 |
| Intensified support | 17 | 2 | (12) | 1.49 | (0.29-7.48) | 0.626 | 15 | 4 | (27) | 1.44 | (0.41-4.97) | 0.567 |
| Special support | 9 | 4 | (44) | 8.96 | (2.07-38.8) | 0.003 | 8 | 3 | (38) | 2.37 | (0.52-10.73) | 0.262 |
| Not known | 20 | 2 | (0) | - |  |  | 17 | 1 | (6) | 0.25 | (0.03-1.97) | 0.187 |
| **Assistant for other reasons at school** |  |  |  |  |  |  |  |  |  |  |  |  |
| No | 183 | 17 | (9) | 1.00 |  |  | 157 | 31 | (20) | 1.00 |  |  |
| Yes | 8 | 2 | (25) | **3.26** | **(0.61-17.4)** | 0.168 | 7 | 3 | (43) | **3.05** | **(0.65-14.3)** | 0.158 |
| **Assistant for T1DM at school** |  |  |  |  |  |  |  |  |  |  |  |  |
| No | 123 | 16 | (13) | 1.00 |  |  | 105 | 26 | (25) | 1.00 |  |  |
| Yes | 68 | 3 | (4) | 0.31 | (0.09-1.10) | 0.070 | 59 | 8 | (14) | 0.48 | (0.20-1.13) | 0.094 |
|  |  |  |  |  |  |  |  |  |  |  |  |  |

* Missing n=1.
